# Emulsifying Properties of Oat Protein/Casein Complex Prepared Using Atmospheric Cold Plasma with pH Shifting

**DOI:** 10.3390/foods14152702

**Published:** 2025-07-31

**Authors:** Yang Teng, Mingjuan Ou, Jihuan Wu, Ting Jiang, Kaige Zheng, Yuxing Guo, Daodong Pan, Tao Zhang, Zhen Wu

**Affiliations:** 1 College of Food Science and Engineering, Ningbo University, Ningbo 315211, China; tengyang2022@163.com (Y.T.); oumingjuan@nbu.edu.cn (M.O.); m19155063132@163.com (T.J.); zhengkaige01@163.com (K.Z.); daodongpan@163.com (D.P.); zhangtao@nbu.edu.cn (T.Z.); 2 Ningbo Enrich Life Bio-Tec Co., Ltd., Ningbo 321004, China; wujihuan@jellybrown.com; 3 School of Food Science & Pharmaceutical Engineering, Nanjing Normal University, Nanjing 210097, China; guoyuxing1981@163.com

**Keywords:** OPI/casein mixtures, atmospheric cold plasma, emulsifying property

## Abstract

An oat protein isolate is an ideal raw material for producing a wide range of plant-based products. However, oat protein exhibits weak functional properties, particularly in emulsification. Casein-based ingredients are commonly employed to enhance emulsifying properties as a general practice in the food industry. pH-shifting processing is a straightforward method to partially unfold protein structures. This study modified a mixture of an oat protein isolate (OPI) and casein by combining a pH adjustment (adjusting the pH of two solutions to 12, mixing them at a 3:7 ratio, and maintaining the pH at 12 for 2 h) with an atmospheric cold plasma (ACP) treatment to improve the emulsifying properties. The results demonstrated that the ACP treatment significantly enhanced the solubility of the OPI/casein mixtures, with a maximum solubility of 82.63 ± 0.33%, while the ζ-potential values were approximately −40 mV, indicating that all the samples were fairly stable. The plasma-induced increase in surface hydrophobicity supported greater protein adsorption and redistribution at the oil/water interface. After 3 min of treatment, the interfacial pressure peaked at 8.32 mN/m. Emulsions stabilized with the modified OPI/casein mixtures also exhibited a significant droplet size reduction upon extending the ACP treatment to 3 min, decreasing from 5.364 ± 0.034 μm to 3.075 ± 0.016 μm. The resulting enhanced uniformity in droplet size distribution signified the formation of a robust interfacial film. Moreover, the ACP treatment effectively enhanced the emulsifying activity of the OPI/casein mixtures, reaching (179.65 ± 1.96 m^2^/g). These findings highlight the potential application value of OPI/casein mixtures in liquid dairy products. In addition, dairy products based on oat protein are more conducive to sustainable development than traditional dairy products.

## 1. Introduction

Rising sustainability concerns have accelerated demand for plant-based protein substitutes [1]. Available sources span cereals (wheat, maize, and rice), legumes (black beans, chickpeas, and lentils), pulses (soybeans and peas), oilseeds (hemp, flax, and sesame), and pseudo-cereals such as quinoa [2]. Oat protein has emerged as a promising candidate due to its substantial protein content (11–24%) and favorable amino acid composition [3]. The absence of key anti-nutrients and allergens [4] further enhances its suitability for food applications, driving global market expansion. Oat protein is increasingly being utilized as a key ingredient in various products, including milk analogs [5], yoghurt [6], cheese [7], and ice cream [8]. Oat protein, primarily a globular structure with a molecular weight of approximately 320 kDa, consists of 54 kDa subunits connected by disulfide bonds [9]. Its globular conformation features abundant surface glutamine residues, resulting in comparatively reduced hydrophilicity relative to other plant proteins [10]. Consequently, oat protein demonstrates limited solubility and foaming capacity, especially within the neutral to slightly acidic pH range (pH 4–7) [10,11]. Dairy proteins like whey and casein dominate emulsion applications. Casein, a principal milk protein accounting for roughly 80% of total milk protein [12], is widely employed in the food sector to boost viscosity, stabilize emulsions and foams, and enhance nutritional profiles [13].

Enhancing protein functionality often involves structural modification, which falls into the following three primary categories: physical, chemical, and biomodification/enzymatic methods. Physical approaches are typically favored for being more sustainable, safer, and operationally simpler than chemical or enzymatic alternatives. Key physical techniques encompass ultrasonic, high-pressure, and cold plasma treatments. Cold plasma, an innovative non-thermal technology, facilitates nano- or microscale structural alterations in materials [14,15]. Atmospheric cold plasma (ACP) comprises a net-neutral mixture of energetic species, such as free electrons, ions, photons, atoms, molecules, and free radicals [16]. Unlike traditional physical modification methods, such as ultrasound and high-pressure homogenization, these particles and reactive chemicals lead to physical and chemical changes in the polymer on the micron to nanometer scale (like protein oxidation). Plasma etching causes protein aggregates and subunits to dissociate, exposing more hydrophobic amino acid residues or peptide chains. Cold plasma technology has been demonstrated to modify plant protein fractions, leading to significant alterations in their technological functionality across diverse proteins like pea [17], grass pea [18], and soybean proteins [19,20]. It has also been reported that cold plasma treatment positively affects the interfacial and emulsification properties of grass pea (*Lathyrus sativus*) protein isolates (GPPIs) in terms of the thermodynamic stabilization of proteins at the interface, globular protein dissociation, and an increase in the surface charge of oil droplets. Cold plasma also enhances plant protein solubility, suggesting its capability of improving the structural and functional properties of oat protein.

pH-shifting processing is a straightforward method to partially unfold protein structures at alkaline or acidic pHs, followed by refolding to a molten globular configuration upon returning to a neutral pH [21]. The partial unfolding of pea proteins and exposure to hydrophobic side chains at a pH of 12 enhance emulsification, improving both emulsifying activity and stability [22]. In addition, pH shifting can promote or exacerbate protein oxidation, but it is not a direct cause of protein oxidation. It mainly increases the sensitivity of proteins to oxidative damage by altering the protein structure (exposing sensitive sites), affecting the production and efficacy of oxidants (particularly metal-catalyzed pathways), and altering the reactivity of key amino acid residues. pH-shifting treatments also positively modify the structure and function of additional plant proteins, including amaranth, peanut [23], and black turtle bean proteins [24].

Studies demonstrate that low-temperature homogenization with casein micelles (CMs) enhances pea protein isolate (PPI) solubility, forming stable co-dispersions [25]. The resulting CM/PPI mixtures show enhanced emulsification and milk-like gelation. Inspired by this, we propose the development of oat/casein complexes using atmospheric pressure cold plasma (ACP) and pH shifting. We further characterize ACP-treated complexes via FTIR, fluorescence spectroscopy, and a particle size distribution analysis to evaluate structural changes. This study combines two different sources of protein, oat protein and casein, and uses pH migration and atmospheric pressure cold plasma (ACP) to improve the functional characteristics of the composite protein, successfully preparing a stable water-in-oil emulsion. The findings elucidate innovative gel-forming mechanisms in engineered protein complexes applicable to plant-based dairy substitutes.

## 2. Materials and Methods

### 2.1. Materials

Casein (80% purity) was purchased from Solarbio Science & Technology Co., Ltd. (Beijing, China). The oat protein isolate (90% purity) was purchased from Shanxi Aite Co., Ltd. (Shanxi, China). Soybean oil was purchased from Fulinmen COFCO (Shanghai, China). Nile red was bought from BBI Life Sciences Co., Ltd. (Shanghai, China). The fluorescein isothiocyanate isomer (FITC) was bought from Macklin Biochemical Co., Ltd. (Shanghai, China). 8-anilino-1-naphthalenesulfonic acid (ANS) and NaOH were bought from Macklin Biochemical Co., Ltd. (Shanghai, China). The PBS buffer and Bradford protein assay kit were purchased from Sangon Biotech Co., Ltd. (Shanghai, China). HCl was bought from Sinopharm Chemical Regant Co., Ltd. (Shanghai, China).

### 2.2. Atmospheric Cold Plasma (ACP) and pH-Shifting Pre-Treatment

Solutions of the oat protein isolate (OPI) and casein (cas) with a protein concentration of 3% (*w*/*v*) were produced to simulate the protein content of milk. Both solutions were pH-adjusted to 12 with 3 mol/L NaOH and magnetically stirred at 1000 rpm for 1 h to ensure complete dissolution. The solutions were mixed at different OPI/cas ratios (0:10, 3:7, 5:5, 7:3, and 10:0) and stirring was continued for 30 min. Finally, the 3:7 group with ACP and pH-shifting treatment was chosen for subsequent experiments because of its highest solubility at a pH of 7 (Figure S1) and relatively large absolute zeta potential values (Figure S2) (the control was a mixed protein solution with pH shifting).

We employed plasma apparatus (Nanjing Prospect Electronics Technology Co., Nanjing, China) featuring a plasma generator, rotating nozzle, and compressor. Sample solutions in beakers underwent treatment while cooled in an ice-water bath and magnetically stirred (800 rpm) with the nozzle positioned 40 mm above the liquid surface. Air served as the working gas (30 L/min) at exposure times of 0, 1, 2, and 3 min. Then, all solutions were pH-adjusted to 7 with 1 mol/L HCl and magnetically stirred at 1000 rpm for 30 min. The treated solution was stored at 4 °C for 12 h for reaction completion before further treatment.

### 2.3. Particle Size Analysis (PSA) and Zeta Potential (ζ-Potential) Estimation

The hydrodynamic radii of the dispersions were measured at 25 °C via dynamic light scattering (Malvern Zetasizer Lab, London, UK) using a 173° backscattering detection angle. Samples were centrifuged and diluted 100 times using distilled water.

The zeta potential was measured using a Zetasizer Lab (Malvern Instruments, Worcestershire, UK) with a DTS1070 capillary cell. Samples were centrifuged and diluted 100 times using distilled water.

### 2.4. Protein Solubility and Surface Hydrophobicity (SHo)

Protein dispersions (30 mg/mL) underwent centrifugation at 11,000 relative centrifugal force (rcf) for 30 min (4 °C), followed by supernatant collection. The soluble protein content in the supernatants was quantified via a Bradford assay [26]. Protein solubility (%) was calculated as (soluble protein content/total protein content) × 100. All analyses were conducted in triplicate.

Protein samples (0.00625–0.1 mg/mL in 10 mM PBS) were prepared following Zhao’s method with modifications [27]. For ANS-binding assays, 4 mL aliquots were mixed with 20 µL of an 8 mM ANS reagent, incubated for 5 min, and analyzed by fluorescence spectroscopy.

### 2.5. Wettability Measurement

Surface wettability and energy were evaluated via contact angle measurements using a DSA100 instrument (KRÜSS, Hamburg, Germany) and the sessile drop technique. This parameter directly correlates with ink adhesion [28]. A 16 μL (determined based on preliminary experiments) drop of distilled water was placed on the surface with a microsyringe and observed through a microscope.

### 2.6. Free Sulfhydryl Content Analysis

The free sulfhydryl group quantification followed Segat’s method [29]. Protein dispersions (5 mg/mL) in 8 M of a Tris-Glycine buffer (10.4 g Tris, 6.9 g glycine, 1.2 g EDTA/L, and a pH of 8.0) were mixed for 25 min at 25 °C. After centrifugation (11,000 rcf, 30 min, and 4 °C), the soluble protein content was quantified via a Bradford assay [26]. For the reaction, 3 mL of the supernatant was combined with 0.02 mL Ellman’s reagent (4 mg/mL DTNB in a buffer). Protein solutions without DTNB served as blanks. Following a 1 h dark incubation (25 °C), absorbance at 412 nm was measured using a TECAN Infinite 200 Pro microplate reader (Männedorf, Switzerland). The free sulfhydryl content (μmol SH/mg) was calculated as follows:

(1) Free SH (μmol SH/mg protein) = (73.53 × A_412_ × D)/C

Here, A denotes absorbance at 412 nm, C is the post-centrifugation sample concentration (mg/mL), and D represents the dilution factor. A coefficient of 73.53 was derived from 10^6^/(13,600 M^−1^ cm^−1^), where 10^6^ accounts for molar-to-mmol/mL and mg/g unit conversions.

### 2.7. FTIR and Intrinsic Fluorescence Analysis

FTIR spectra (4000–400 cm^−1^) of control and treated samples were acquired using a Nicolet iS20 spectrometer (Thermo Scientific, Waltham, MA, USA) at a 4 cm^−1^ resolution with 64 cumulative scans. Fluorescence emission profiles (220–600 nm) were recorded on an F-4700 spectrophotometer (Hitachi, Tokyo, Japan) equipped with a xenon lamp, 1.0 cm quartz cells, and a thermostat-controlled sample chamber. The excitation wavelengths were set to 280 nm (the excitation wavelengths were set to 280 nm because this corresponds with the main chromogenic amino acids tryptophan and tyrosine). Excitation and emission slit widths were fixed at 5 nm, with a scan speed of 12,000 nm/min, and the PMT voltage was set to 450 V.

### 2.8. Dynamic Interfacial Pressure

Interfacial tension dynamics (σ vs. adsorption time t) during OPI/casein adsorption at the oil–water interface were monitored using a DSA25E video optical contact angle system (KRÜSS, Germany). Prior to testing, the microsyringe and needle were pre-rinsed with a sample solution. A glass tank filled with soybean oil (ρ = 0.920 g/cm^3^) was positioned beneath the syringe, with the needle tip immersed in the oil, and the system was sealed during operation. Protein solution droplets (30 μL) were delivered to the oil phase via the needle. Upon interface formation, the droplet contours were continuously imaged while recording the surface tension. Dynamic interfacial pressure (π) was calculated as π = σ_0_ − σ_t_, where σ_0_ = 18.69 mN/m (buffer–soybean oil interfacial tension) and σ_t_ = tension at time t. The measurements were conducted with minimized ambient light and mechanical vibration.

### 2.9. Preparation of O/W Emulsions

The aqueous phase (at an OPI/casein concentration of 22 mg/mL, determined by the Bradford method) and soybean oil phase were mixed at a volume ratio of 4:1. Using a high-shear homogenizer (XHF-DY, Scientz, Ningbo, China), emulsions were prepared through intermittent homogenization, as follows: 6 cycles of 20 s processing at 10,000 rpm, totaling 2 min. This emulsion could be further processed and applied in the food industry, such as by encapsulating nutrient-active substances or fermentation to produce fermented milk products.

### 2.10. Measurement of Emulsion Droplet Size and EAI of O/W Emulsions

The emulsion droplet size distribution was characterized via laser diffraction (BT-9300S analyzer, Baite, Dandong, China), adapting Gao’s methodology with modifications [30]. The results reported the volume-weighted mean diameter (D [4,3]) and size distribution profile.

The emulsification activity index (EAI) was quantified following Li’s method with modifications [31]. Post-homogenization, 50 μL aliquots were collected from the emulsion bottom phase and diluted in 5 mL of a 0.1% SDS solution. After vortex mixing, absorbance at 500 nm (A_500_) was measured using a microplate reader. The EAI was calculated as follows:

(2) EAI (m^2^/g) = (4.606 × A × dilution factor)/[c × φ × (1 − θ) × 10^4^]

For a fixed dilution factor of 100, c denotes the protein’s initial concentration (mg/mL), φ is the optical path length (0.01 m), and θ is the oil fraction in the emulsion formulation (20%). A is the absorbance of the diluted emulsion.

### 2.11. Morphology of Emulsion Droplets

Emulsion droplet morphology was analyzed by confocal laser scanning microscopy (Leica SP8 X, Wetzlar, Germany) following established protocols [32]. For dual-channel staining, emulsions were mixed with equivolume fluorescent dyes (0.5% FITC and 0.2% Nile red, *w*/*v*) at 1:50 (*v*/*v*). After brief vortexing, the samples were mounted on slides. Protein and lipid phases were specifically labeled using FITC (excitation at 488 nm) and Nile red (excitation at 543 nm), respectively, using a 40× objective lens.

### 2.12. Statistical Analysis

Statistical significance was assessed via a one-way ANOVA using SPSS (version 25), with *p* < 0.05 indicating significant differences. The Duncan test was used to compare the means. All values were expressed as the mean ± standard deviation (X ± SD).

## 3. Results

### 3.1. Protein Solubility, ζ-Potential, and Particle Size Distribution of OPI/Casein

**Figure 1:**
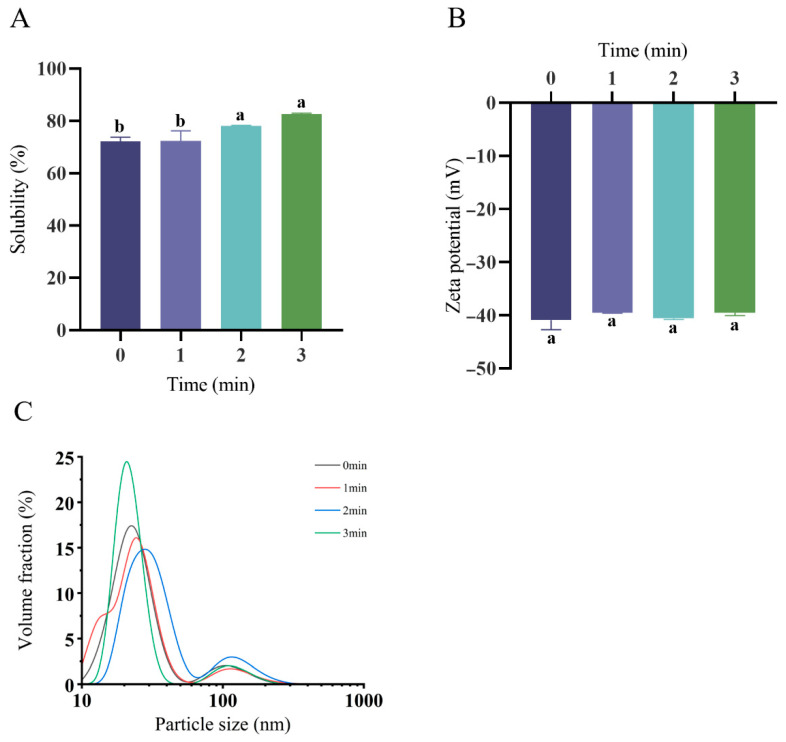
Physical properties of the OPI/casein solution mixtures upon ACP treatment. Effect of OPI/casein with and without ACP treatment on the solubility (**A**), zeta potential (**B**), and particle size distribution (**C**). Different letters indicate significant differences (*p* < 0.05) among the groups.

Figure 1A demonstrates the enhanced protein solubility after the plasma treatment (82.63 ± 0.33% at 3 min) versus untreated controls (72.17 ± 1.66%), representing a 14.49% increase. This elevation correlates with cold plasma oxidation generating hydrophilic moieties (e.g., carbonyl/hydroxyl groups). Corresponding ζ-potential values appear in Figure 1B. The zeta potential values for the 0 min, 1 min, 2 min, and 3 min groups were −40.96 ± 1.76 mV, −39.58 ± 0.05 mV, −40.61 ± 0.19 mV, and −40.18 ± 0.96 mV, respectively. There were no significant differences in the zeta potential values between the native and plasma-treated samples (*p* = 0.418; *p* > 0.05). Figure 1C and Table 1 characterize the particle size distributions for native and plasma-treated OPI/casein dispersions, revealing consistent bimodality. Plasma processing induced significant particle enlargement versus the controls. Progressive peak right-shifting at 1–2 min treatment durations confirmed the size expansion. Conversely, the 3 min treatment triggered left-shifting (Figure 1C), suggesting partial size reduction, although the mean diameters remained elevated relative to the untreated samples.

### 3.2. Free Sulfhydryl, FTIR, and Surface Hydrophobicity Analyses

**Figure 2:**
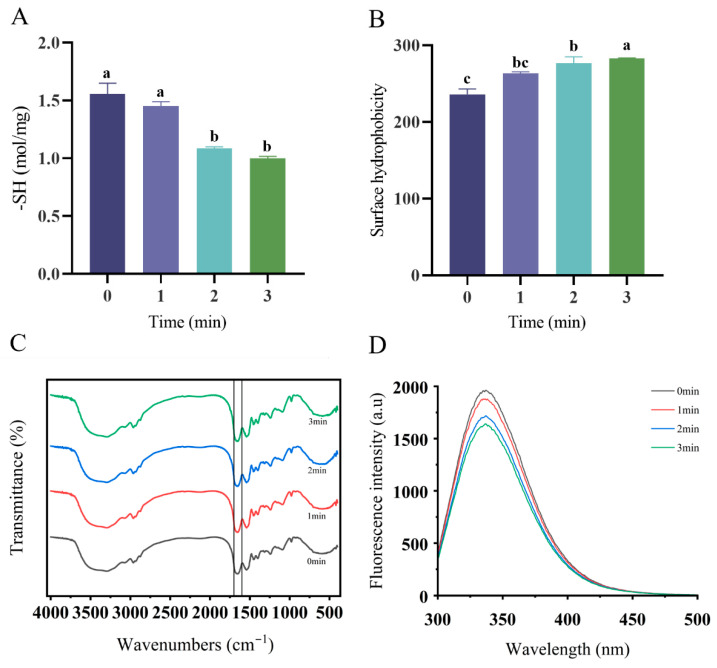
The structural changes in the OPI/casein solution mixtures upon ACP treatment. Effect of OPI/casein with and without ACP treatment on the free sulfhydryl groups (**A**), surface hydrophobicity (**B**), FTIR (**C**), and intrinsic fluorescence (**D**). Different letters indicate significant differences (*p* < 0.05) among the groups.

The control samples maintained maximal free sulfhydryl content, as shown in Figure 2A. Plasma processing progressively depleted these groups, demonstrating pronounced time-dependent reduction effects (*p* = 0.001; *p* < 0.05). These free radicals are unstable and easily form disulfide bonds (-S-S-) within or between molecules, thereby strengthening the bonds between protein molecules and increasing the stability of a complex. The surface hydrophobicity (SHo) of the protein complexes, quantified by ANS-binding fluorescence (Figure 2B), demonstrated a time-dependent escalation with plasma exposure. The SHo values surged from 236.12 ± 7.13 RIF (Relative Fluorescence Intensity) to 282.65 ± 0.77 RIF as the treatment duration increased, confirming progressive hydrophobic enhancement. An increase in surface hydrophobicity is associated with protein unfolding. In general, surface hydrophobicity and solubility are negatively correlated, but in this study, protein solubility also increased, which may be due to the fact that the protein unfolding process also exposed more hydrophilic groups.

The FTIR spectra of oat protein (Figure 2C) revealed key functional groups via a peak intensity analysis. Plasma-induced structural alterations manifested without peak position shifts. Characteristic absorptions included 1453/1398 cm^−1^ methyl/methylene groups; 1540 cm^−1^ (amide II) N-H stretching; and 1659 cm^−1^ (amide I) C=O vibrations.

Hydroxyl-mediated oxidation reduced aromatic amino acid peaks (3060–3090 cm^−1^). Carbonylation was confirmed by elevated carbonyl peak intensity in the treated complexes. Amide I band intensification was also observed.

The UV spectra (Figure 2D) showed the control λ_max_ at 337 nm. Plasma treatment induced hypsochromic shifts to 336.6 nm (1 min), 336.8 nm (2 min), and 336.4 nm (3 min).

**Figure 3:**
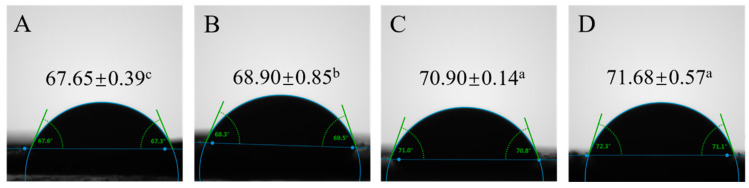
Wettability analysis of the OPI/casein mixtures with different ACP treatments. Contact angle of OPI/casein with treatment time: 0 min (**A**), 1 min (**B**), 2 min (**C**), and 3 min (**D**). Different letters indicate significant differences (*p* < 0.05) among the groups.

Figure 3 demonstrates that the untreated (0 min) mixed protein surfaces displayed hydrophilic characteristics, evidenced by a contact angle of 67.65° ± 0.39°. Following 3 min of plasma exposure, this value rose significantly to 71.68° ± 0.57°, indicating enhanced surface hydrophobicity (*p* = 0.000; *p* < 0.05). This hydrophobic transition arises from plasma-induced structural alterations. Through plasma etching, reversible protein aggregates dissociate into subunits, exposing buried hydrophobic residues and peptide chains. The resulting enhancement in mixed protein hydrophobicity corroborates our surface hydrophobicity measurements.

### 3.3. Secondary Structure of OPI/Casein

The quantitative secondary structural changes in plasma-treated OPI/casein complexes are detailed in Table 2. Following 3 min plasma exposure, the α-helix content increased by 0.64% (12.16% → 12.80%), β-sheet decreased by 1.34% (28.29% → 26.95%), β-turn rose by 0.97% (42.16% → 43.13%), and random coil diminished by 0.29% (17.40% → 17.11%).

These conformational shifts originated from reactive-species-mediated peptide oxidation. Notably, the enhanced α-helical fraction (12.80%) signified improved structural stability and order versus the native proteins.

### 3.4. Emulsion Droplet Size Distribution and Emulsification Activity

**Figure 4:**
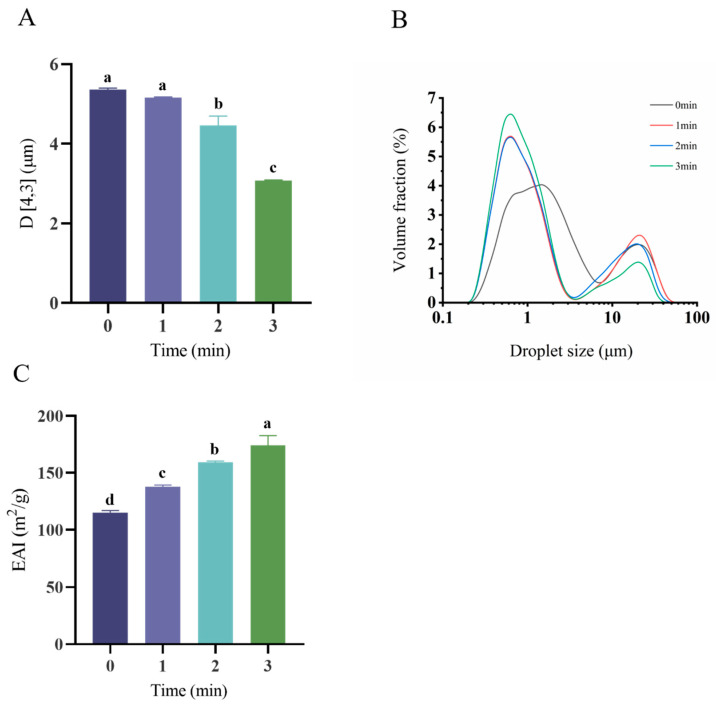
Emulsion droplet size and distribution analysis of the OPI/casein solution. Effect of OPI/casein with and without ACP treatment on the emulsion droplet size (D [4,3]) (**A**), emulsion droplet size distribution (**B**), and EAI (**C**). Different letters indicate significant differences (*p* < 0.05) among the groups.

Emulsion droplet size distribution and volumetric mean diameter (D [4,3]) critically govern emulsion functionality, as depicted in Figure 4A,B. All systems displayed characteristic bimodal distribution profiles, although peak positions and amplitudes varied significantly with plasma exposure duration. Specifically, the droplet dimensions exhibited pronounced time-dependent modulation under plasma processing (*p* = 0.000; *p* < 0.05). Emulsions stabilized by native OPI/casein complexes exhibited a volumetric mean diameter of 5.364 ± 0.034 μm (Figure 4A). A progressive reduction in droplet dimensions occurred with an extended plasma exposure duration, culminating in a significantly diminished average size of 3.075 ± 0.016 μm following the 3 min treatment. This reduction suggests the formation of a thick and elastic interfacial film around the oil droplets induced by the rise of the surface hydrophobicity. The emulsification activity index (EAI) is presented in Figure 4C. The EAI significantly increased with a prolonged plasma treatment time (*p* = 0.000; *p* < 0.05). This phenomenon corresponded with the result above. This is due to the fact that proteins unfold after plasma treatment and more hydrophilic and hydrophobic groups are exposed, resulting in increased protein amphiphilicity.

### 3.5. CLSM and Interfacial Property Analyses

**Figure 5:**
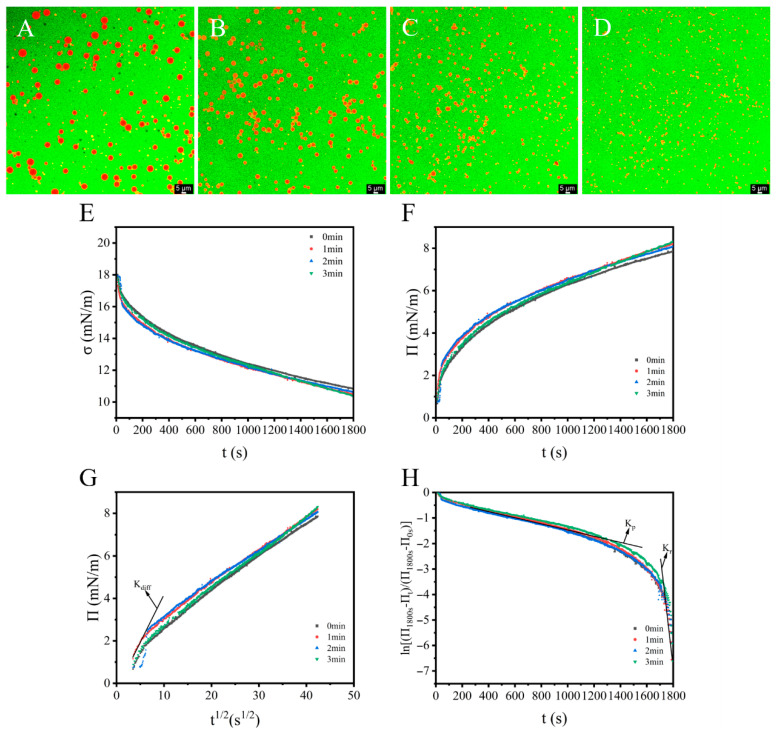
Morphology of emulsions stabilized by OPI/casein with different ACP treatment times. Treatment times of 0 min (**A**), 1 min (**B**), 2 min (**C**), and 3 min (**D**). (**E**) Temporal evolution of interfacial tension (σ). (**F**) Interfacial pressure (π) kinetics over time. (**G**) Linear relationship between t 1/2 and π for diffusion rate constant k_diff_ determination of time dependence of ln [(π1800s − πt)/(π1800s − π0s)] for penetration (k_p_) and rearrangement rate constant (k_r_) (**H**).

CLSM imaging elucidated the microstructural evolution of OPI/casein-stabilized emulsions, revealing significant plasma-dependent alterations in the droplet morphology and size distribution. Pre-treatment emulsions (Figure 5A) displayed large, polydisperse droplets. Progressive plasma exposure induced a substantial size reduction and distribution homogenization (Figure 5B–D), correlating with the laser diffraction data.

An interfacial dynamics analysis (Figure 5E,F) demonstrated rapid π elevation within the initial 45 s, indicating accelerated OPI/casein adsorption at oil–water interfaces. This facilitated a gradual interfacial concentration buildup. The kinetic profiles revealed linear π-t^1/2^ relationships terminating before 6 s^1/2^ (Figure 5G). Diffusion coefficients (k_diff_; Table 3) derived from linear regression slopes confirmed enhanced interfacial diffusion in the plasma-treated groups versus the controls, except for the 3 min samples, which exhibited marginally reduced diffusion rates. This time-dependent behavior reflects the plasma-induced structural rearrangements in protein complexes.

**Table 3:** The interfacial pressure at 1800 s (π_1800s_), diffusion rate (*k_diff_*), penetration rate (*k_p_*) and rearrangement rate (*k_r_*) constant of OPI/Casein at O/W interface with and without ACP treatment.

	π1800s (mN/m)	kdiff (mN/m/s1/2)	kp (×103/s)	kr (×103/s)
0 min	7.86	0.427	1.00	66.00
1 min	8.19	0.437	1.00	79.00
2 min	8.08	1.36	1.00	56.00
3 min	8.32	0.424	1.00	92.00

The adequate diffusion and accumulation of OPI/casein at the interface (Figure 5F) may account for the observed consistency in structural elongation, resulting in similar kp values. The values of kr were significantly higher than those of kp, indicating that the structural rearrangement of the OPI/casein mixtures played a more substantial role in adsorption and interfacial formation than their permeation activity. Compared with the control, all plasma-treated groups exhibited higher kr values, apart from the group subjected to 2 min of plasma treatment, likely due to the larger particle size in that sample.

## 4. Discussion

Protein solubility governs the technological functionality of plant proteins in formulated foods—particularly modified dairy analogs—by directly modulating their physicochemical behavior. Key solubility determinants encompass the pH environment, surface charge distribution, particle dimensions, and hydrophilic/hydrophobic residue balance. Surface hydrophobicity (SHo), intrinsically linked to protein conformational states and solubility [33], increased after the plasma treatment. This hydrophobic shift stems from the plasma-etching-induced dissociation of reversible protein aggregates into subunits, exposing buried hydrophobic domains.

Plasma processing exposes spatially sequestered hydrophobic residues and peptide chains buried at protein domain/subdomain interfaces or oligomeric subunit junctions [34,35]. This mechanism aligns with Segat et al.’s [29] observations in cold-plasma-treated whey protein isolates.

Contact angle (θ) quantification provides critical insights into modified materials’ surface wettability. In our study, the 3 min plasma-treated samples achieved θ ≈ 90° (Figure 3), indicating a markedly enhanced oil–water interfacial adsorption capacity [36] that could promote interfacial recruitment [37].

Cold-plasma-derived reactive oxygen/nitrogen species (RONS) mediate oxidative modifications to protein micelles, unmasking buried active sites. This structural rearrangement enhances hydrophilic interactions with aqueous phases, consequently elevating solubility—a phenomenon corroborated by Gunaseelan [1], wherein plasma-treated oat proteins universally demonstrated superior solubility versus controls. Concurrently, hydrodynamic diameter expansion in treated samples reflects probable plasma-induced aggregation pathways. This is not contradictory to the increase in solubility, and may be due to the fact that the increase in oxygen-containing groups on the surface of the protein after plasma treatment has a more pronounced effect on the increase in solubility. Microscopic evidence of particle aggregation in plasma-decontaminated oat milk substantiates this mechanism. Convergent findings across cold-plasma-modified plant proteins—including pea isolates [17], flaxseed [38], oat [1,2], and soybean isolates [19,20]—validate our experimental outcomes. Protein aggregation arises from intramolecular interactions governed by surface electrostatic potentials and hydrophobic forces. Consequently, zeta potential measurements were excluded from core analyses, consistent with Sharma and Singh [39] null findings in milk treated for ≤ 5.5 min. Wang et al. corroborated this observation, reporting insignificant zeta potential alterations in plasma-treated sheep’s milk within 5 min [40].

Cold plasma modification induces multifaceted alterations in sulfur-containing amino acid side chains. The primary detectable transformation involves sulfhydryl group oxidation to disulfides and derived sulfur species [41]. Concomitantly, sulfhydryl radicals react with molecular oxygen, generating thiol peroxyl radicals that subsequently form disulfide bonds through thiol group interactions [41].

Ozone—abundantly produced during plasma discharge—critically mediates cysteine oxidation, converting thiol groups (-SH) to disulfides, with consequent free thiol depletion [42,43]. Crucially, hydroxyl radicals (•OH) exhibit 5-fold greater reactivity than ozone in thiol oxidation kinetics [44]. This differential reactivity establishes •OH as the predominant contributor to free SH reduction during plasma processing.

The effect of sulfhydryl oxidation on protein aggregation and solubility is dual and requires balancing disulfide bond formation with protein stability by regulating oxidative conditions to achieve target functions.

Fluorescence spectroscopy sensitively probes tertiary protein conformational shifts during denaturation, unfolding, and aggregation [35]. Endogenous fluorophores—tryptophan (Trp), tyrosine (Tyr), and phenylalanine (Phe)—generate intrinsic fluorescence signals [45]. Spectral parameters (FI_max_ and λ_max_) directly report the spatial positioning of aromatic residues within protein 3D architectures [34,35].

Trp/Tyr exhibit peak excitation at 280 nm, whereas Phe contributes minimally due to a low quantum yield [45]. A λ_max_ blue shift signifies reduced local polarity around fluorophores, reflecting hydrophobic microenvironments [19,20]. The subsequent exposure of non-polar residues drives aggregation via hydrophobic clustering [46].

Maximum fluorescence intensity (FI) progressively diminishes with extended plasma exposure, exhibiting a 4.03–16.19% reduction range. This attenuation primarily originates from the hydroxyl-radical-mediated oxidation of tryptophan/tyrosine residues via hydroxyl group incorporation [43,47]. Concurrent fluorescence quenching—driven by protein molecular aggregation and the consequent burial of aromatic fluorophores through hydrophobic clustering—further contributes to FI decline [14,48,49].

Electron transfer from excited-state tryptophan (Trp) indole rings to plasma-generated carbonyl groups induces intramolecular Trp fluorescence quenching [50,51]. Extended treatment durations intensify oxidation-mediated carbonyl accumulation, exacerbating fluorescence attenuation [18].

Plasma-induced structural unfolding in OPI/casein complexes enhance hydrophobic domain exposure, facilitating their adsorption and interfacial redistribution at oil/water interfaces [52,53] despite concurrent increases in overall protein particle dimensions.

The plasma-induced elevation in surface hydrophobicity critically facilitates these interfacial modifications. Extensive evidence confirms that heightened protein surface hydrophobicity accelerates adsorption kinetics and spatial reorganization at oil/water interfaces [52,53]. This promotes the development of coherent viscoelastic layers that mechanically impede droplet flocculation and coalescence through enhanced interfacial steric stabilization. This occurs because increased protein surface hydrophobicity enhances interfacial adsorption by strengthening hydrophobic interactions, reducing interfacial tension, and facilitating conformational adaptation. In addition, the effect of increasing surface hydrophobicity on antioxidant properties is two-fold; a moderate increase in surface hydrophobicity enhances the antioxidant properties, whereas an excessive increase, resulting in protein aggregation, weakens the antioxidant properties. Therefore, a moderate modification is needed. All plasma-modified samples exhibited elevated interfacial adsorption equilibria (π1800s) relative to the untreated controls, with the 3 min treatment group demonstrating maximal enhancement. This adsorption behavior directly correlates with the augmented surface hydrophobicity profiles documented in Figure 2B.

Protein adsorption proceeds through the following three sequential phases:

(I) Diffusion: Initial adsorption is driven by concentration gradients, exhibiting linear π vs. t 1/2 kinetics when diffusion-controlled. The slope defines the diffusion coefficient (k_diff_) [54].
(II) Penetration and (III) Rearrangement: Post-diffusion barriers (interfacial steric constraints/energy barriers) dominate, transitioning adsorption kinetics to penetration–rearrangement dependence [55]. This multi-stage behavior is quantifiable via first-order phenomenological modeling [41].
  (3) ln[(π1800s − πt)/(π1800s − π0s)] = − ki × t

Here, π1800s, πt, and π0s are, respectively, π at the final time (1800 s), at any time (t), and at the initial time (when π starts to appear), and ki is a first-order rate constant. Typically, the resulting plot from this equation reveals two distinct linear regions. The first slope of the adsorption plot is referred to as the permeability (kp), while the second slope represents the rearrangement rate (kr). No significant differences were observed among the groups (Table 3), indicating that plasma treatment had a minimal impact on the penetration and interfacial unfolding of OPI/casein mixtures [56] despite its effects on the size and distribution of the OPI/casein mixtures in the bulk aqueous phase, which revealed that the plasma process enhanced the stability of the OPI/casein mixtures.

## 5. Conclusions

Synergistic pH shifting and atmospheric cold plasma (ACP) processing significantly improved the solubility and emulsification functionality in oat protein isolate (OPI)/casein complexes. ACP exposure induced structural reorganization, unmasking buried hydrophobic domains and elevating surface hydrophobicity. These modifications promoted robust interfacial adsorption, facilitating the development of cohesive viscoelastic layers encapsulating oil droplets. Collectively, the combined treatment modulated OPI/casein conformation to optimize the emulsifying performance, establishing applicability as a novel emulsifier in liquid food matrices. Finally, our research found that plasma treatment requires an appropriate duration and intensity, otherwise it may have negative effects (such as protein cleavage).

## Figures and Tables

**Table 1:** Particle size of OPI/casein with and without ACP treatment.

Treatment Time (min)	0	1	2	3
Particle size (nm)	149.70 ± 1.73 ^c^	157.40 ± 2.85 ^b^	178.30 ± 1.13 ^a^	151.96 ± 0.99 ^c^

Different letters indicate significant differences (*p* < 0.05) among the groups.

**Table 2:** Secondary structure of OPI/casein with and without ACP treatment.

Sample	Alpha Helix (%)	Beta Sheet (%)	Turn (%)	Unordered Coil (%)
0 min	12.16	28.29	42.16	17.40
1 min	12.46	28.27	41.75	17.51
2 min	12.75	28.18	42.16	16.91
3 min	12.80	26.95	43.13	17.12

## Data Availability

The original contributions presented in the study are included in the article/Supplementary Materials, further inquiries can be directed to the corresponding author.

## Supplementary Materials

The following supporting information can be downloaded at: https://www.mdpi.com/article/10.3390/foods14152702/s1. Figure S1: Effect of plasma treatment on the solubility of OPI/casein at different ratios. Different letters indicate significant differences (*p* < 0.05) among the groups. Figure S2: Effect of plasma treatment on the zeta potential of OPI/casein at different ratios. Different letters indicate significant differences (*p* < 0.05) among the groups.

## References

[1] Eazhumalai G., Kalaivendan R.G.T., Annapure U.S. (2023). Effect of atmospheric pin-to-plate cold plasma on oat protein: Structural, chemical, and foaming characteristics. Int. J. Biol. Macromol..

[2] Eazhumalai G., Ranjitha Gracy T.K., Mishra A., Annapure U.S. (2021). Atmospheric pressure nonthermal pin to plate plasma system for the microbial decontamination of oat milk. J. Food Process. Pres..

[3] Zhao C.-B., Zhang H., Xu X.-Y., Cao Y., Zheng M.-Z., Liu J.-S., Wu F. (2017). Effect of acetylation and succinylation on physicochemical properties and structural characteristics of oat protein isolate. Process Biochem..

[4] Mollakhalili-Meybodi N., Arab M., Zare L. (2022). Harmful compounds of soy milk: Characterization and reduction strategies. J. Food Sci. Technol..

[5] Paul A.A., Kumar S., Kumar V., Sharma R. (2020). Milk Analog: Plant based alternatives to conventional milk, production, potential and health concerns. Crit. Rev. Food Sci. Nutr..

[6] Brückner-Gühmann M., Benthin A., Drusch S. (2019). Enrichment of yoghurt with oat protein fractions: Structure formation, textural properties and sensory evaluation. Food Hydrocoll..

[7] Hamdy S.M., Hassan M.G., Ahmed R.B., Abdelmontaleb H.S. (2021). Impact of oat flour on some chemical, physicochemical and microstructure of processed cheese. J. Food Process. Pres..

[8] Salem S.A., Hamad E.M., Ashoush I.S. (2016). Effect of Partial Fat Replacement by Whey Protein, Oat, Wheat Germ and Modified Starch on Sensory Properties, Viscosity and Antioxidant Activity of Reduced Fat Ice Cream. FNS.

[9] Li Y., Zeng Q.-H., Liu G., Peng Z., Wang Y., Zhu Y., Liu H., Zhao Y., Jing Wang J. (2021). Effects of ultrasound-assisted basic electrolyzed water (BEW) extraction on structural and functional properties of Antarctic krill (*Euphausia superba*) proteins. Ultrason. Sonochem..

[10] Amagliani L., Silva J.V.C., Saffon M., Dombrowski J. (2021). On the foaming properties of plant proteins: Current status and future opportunities. Trends Food Sci. Tech..

[11] Kumar L., Sehrawat R., Kong Y. (2021). Oat proteins: A perspective on functional properties. LWT.

[12] Zong M., Tong X., Farid M.S., Chang C., Guo Y., Lian L., Zeng X., Pan D., Wu Z. (2023). Enhancement of gum Arabic/casein microencapsulation on the survival of *Lactiplantibacillus plantarum* in the stimulated gastrointestinal conditions. Int. J. Biol. Macromol..

[13] Ranadheera C.S., Liyanaarachchi W.S., Chandrapala J., Dissanayake M., Vasiljevic T. (2016). Utilizing unique properties of caseins and the casein micelle for delivery of sensitive food ingredients and bioactives. Trends Food Sci. Technol..

[14] Dong S., Gao A., Xu H., Chen Y. (2017). Effects of Dielectric Barrier Discharges (DBD) Cold Plasma Treatment on Physicochemical and Structural Properties of Zein Powders. Food Bioprocess. Technol..

[15] Ekezie F.-G.C., Cheng J.-H., Sun D.-W. (2019). Effects of atmospheric pressure plasma jet on the conformation and physicochemical properties of myofibrillar proteins from king prawn (*Litopenaeus vannamei*). Food Chem..

[16] Feizollahi E., Arshad M., Yadav B., Ullah A., Roopesh M. (2020). Degradation of deoxynivalenol by atmospheric-pressure cold plasma and sequential treatments with heat and UV light. Food Eng. Rev..

[17] Bu F., Nayak G., Bruggeman P., Annor G., Ismail B.P. (2022). Impact of plasma reactive species on the structure and functionality of pea protein isolate. Food Chem..

[18] Mahdavian Mehr H., Koocheki A. (2020). Effect of atmospheric cold plasma on structure, interfacial and emulsifying properties of Grass pea (*Lathyrus sativus* L.) protein isolate. Food Hydrocoll..

[19] Zhang Q., Cheng Z., Zhang J., Nasiru M.M., Wang Y., Fu L. (2021). Atmospheric cold plasma treatment of soybean protein isolate: Insights into the structural, physicochemical, and allergenic characteristics. J. Food Sci..

[20] Zhang S., Huang W., Feizollahi E., Roopesh M.S., Chen L. (2021). Improvement of pea protein gelation at reduced temperature by atmospheric cold plasma and the gelling mechanism study. Innov. Food Sci. Emerg. Technol..

[21] Zhang S., Huang W., Roopesh M.S., Chen L. (2022). Pre-treatment by combining atmospheric cold plasma and pH-shifting to prepare pea protein concentrate powders with improved gelling properties. Food Res. Int..

[22] Jiang J., Zhu B., Liu Y., Xiong Y.L. (2014). Interfacial Structural Role of pH-Shifting Processed Pea Protein in the Oxidative Stability of Oil/Water Emulsions. J. Agr. Food Chem..

[23] Li J., Wu M., Wang Y., Li K., Du J., Bai Y. (2020). Effect of pH-shifting treatment on structural and heat induced gel properties of peanut protein isolate. Food Chem..

[24] He S., Zhao J., Cao X., Ye Y., Wu Z., Yue J., Yang L., Jin R., Sun H. (2020). Low pH-shifting treatment would improve functional properties of black turtle bean (*Phaseolus vulgaris* L.) protein isolate with immunoreactivity reduction. Food Chem..

[25] Krentz A., García-Cano I., Ortega-Anaya J., Jiménez-Flores R. (2022). Use of casein micelles to improve the solubility of hydrophobic pea proteins in aqueous solutions via low-temperature homogenization. J. Dairy Sci..

[26] Bradford M.M. (1976). A rapid and sensitive method for the quantitation of microgram quantities of protein utilizing the principle of protein-dye binding. Anal. Biochem..

[27] Zhao X.L., Zhao Q., Chen H.B., Xiong H. (2019). Distribution and effects of natural selenium in soybean proteins and its protective role in soybean β-conglycinin (7S globulins) under AAPH-induced oxidative stress. Food Chem..

[28] Dixon D., Meenan B.J. (2012). Atmospheric Dielectric Barrier Discharge Treatments of Polyethylene, Polypropylene, Polystyrene and Poly(ethylene terephthalate) for Enhanced Adhesion. J. Adhes. Sci. Technol..

[29] Segat A., Misra N.N., Cullen P.J., Innocente N. (2015). Atmospheric pressure cold plasma (ACP) treatment of whey protein isolate model solution. Innov. Food Sci. Emerg. Technol..

[30] Gao H., Cheng C., Fang S., McClements D.J., Ma L., Chen X., Zou L., Liang R., Liu W. (2022). Study on curcumin encapsulated in whole nutritional food model milk: Effect of fat content, and partitioning situation. J. Funct. Foods.

[31] Li R., True A.D., Sha L., Xiong Y.L. (2024). Structural modification of oat protein by thermosonication combined with high pressure for O/W emulsion and model salad dressing production. Int. J. Biol. Macromol..

[32] Bai Y., Zeng X., Zhang C., Zhang T., Wang C., Han M., Zhou G., Xu X. (2021). Effects of high hydrostatic pressure treatment on the emulsifying behavior of myosin and its underlying mechanism. LWT.

[33] Chang C., Tu S., Ghosh S., Nickerson M.T. (2015). Effect of pH on the inter-relationships between the physicochemical, interfacial and emulsifying properties for pea, soy, lentil and canola protein isolates. Food Res. Int..

[34] Mohseni-Shahri F.S., Housaindokht M.R., Bozorgmehr M.R., Moosavi-Movahedi A.A. (2014). The influence of the flavonoid quercetin on the interaction of propranolol with human serum albumin: Experimental and theoretical approaches. J. Lumin..

[35] Li J., Xiang Q., Liu X., Ding T., Zhang X., Zhai Y., Bai Y. (2017). Inactivation of soybean trypsin inhibitor by dielectric-barrier discharge (DBD) plasma. Food Chem..

[36] Sun G., Liu X., McClements D.J., Liu S., Li B., Li Y. (2021). Chitin nanofibers improve the stability and functional performance of Pickering emulsions formed from colloidal zein. J. Colloid Interface Sci..

[37] Liu Z., Lin D., Shen R., Yang X. (2021). Bacterial cellulose nanofibers improved the emulsifying capacity of soy protein isolate as a stabilizer for pickering high internal-phase emulsions. Food Hydrocoll..

[38] Yu X., Huang S., Nie C., Deng Q., Zhai Y., Shen R. (2020). Effects of atmospheric pressure plasma jet on the physicochemical, functional, and antioxidant properties of flaxseed protein. J. Food Sci..

[39] Sharma S., Singh R.K. (2022). Effect of atmospheric pressure cold plasma treatment time and composition of feed gas on properties of skim milk. LWT.

[40] Wang D., Zhang Z., Yang X., Zhang Y., Li Y., Zhao Y. (2021). Multi-scenario simulation on the impact of China’s electricity bidding policy based on complex networks model. Energ. Policy.

[41] Zhao T., Zhao Y., Nitta Y., Yagioka A., Komatsuzaki M. (2013). Performance of a No-tillage Seeder with Different Cover Crop Species and Residue Management for Sweet Sorghum for Sustainable Biofuel Production. Eng. Agric. Environ. Food.

[42] Tang C.-H., Sun X. (2010). Physicochemical and Structural Properties of 8S and/or 11S Globulins from Mungbean [*Vigna radiata* (L.) Wilczek] with Various Polypeptide Constituents. J. Agric. Food Chem..

[43] Takai E., Kitamura T., Kuwabara J., Ikawa S., Yoshizawa S., Shiraki K., Kawasaki H., Arakawa R., Kitano K. (2014). Chemical modification of amino acids by atmospheric-pressure cold plasma in aqueous solution. J. Phys. D Appl. Phys..

[44] Shin D.N., Park C.W., Hahn J.W. (2000). Detection of OH(A2σ+) and O(1D) emission spectrum generated in a pulsed corona plasma. BKCS.

[45] Bußler S., Rumpold B.A., Fröhling A., Jander E., Rawel H.M., Schlüter O.K. (2016). Cold atmospheric pressure plasma processing of insect flour from Tenebrio molitor: Impact on microbial load and quality attributes in comparison to dry heat treatment. Innov. Food Sci. Emerg. Technol..

[46] Ji H., Han F., Peng S., Yu J., Li L., Liu Y., Chen Y., Li S., Chen Y. (2019). Behavioral Solubilization of Peanut Protein Isolate by Atmospheric Pressure Cold Plasma (ACP) Treatment. Food Bioprod. Process.

[47] Zhou R., Zhou R., Zhuang J., Zong Z., Zhang X., Liu D., Bazaka K., Ostrikov K. (2016). Interaction of Atmospheric-Pressure Air Microplasmas with Amino Acids as Fundamental Processes in Aqueous Solution. PLoS ONE.

[48] Dong S., Gao A., Zhao Y., Li Y.-t., Chen Y. (2017). Characterization of physicochemical and structural properties of atmospheric cold plasma (ACP) modified zein. Food Bioprod. Process.

[49] Dong S., Wang J.-m., Cheng L.-m., Lu Y.-l., Li S.-h., Chen Y. (2017). Behavior of Zein in Aqueous Ethanol under Atmospheric Pressure Cold Plasma Treatment. J. Agric. Food Chem..

[50] Alston R.W., Lasagna M., Grimsley G.R., Scholtz J.M., Reinhart G.D., Pace C.N. (2008). Peptide Sequence and Conformation Strongly Influence Tryptophan Fluorescence. Biophys. J..

[51] Callis P.R., Liu T. (2004). Quantitative Prediction of Fluorescence Quantum Yields for Tryptophan in Proteins. J. Phys. Chem. B.

[52] Mundo J.L.M., Zhou H., Tan Y., Liu J., McClements D.J. (2021). Enhancing emulsion functionality using multilayer technology: Coating lipid droplets with saponin-polypeptide-polysaccharide layers by electrostatic deposition. Food Res. Int..

[53] Hu Y., Zhou C., Du L., Zhan F., Sun Y., Wu Z., Pan D. (2023). Phenolic structure dependent interaction onto modified goose liver protein enhanced by pH shifting: Modulations on protein interfacial and emulsifying properties. Int. J. Biol. Macromol..

[54] Perez A.A., Carrara C.R., Sánchez C.C., Santiago L.G., Rodríguez Patino J.M. (2009). Interfacial dynamic properties of whey protein concentrate/polysaccharide mixtures at neutral pH. Food Hydrocoll..

[55] Ma J., Pan C., Chen H., Chen Y., Chen W., Pei J., Zhang M., Zhong Q., Chen W. (2023). Interfacial behavior of coconut (*Cocos nucifera* L.) globulins at different pH: Relation to emulsion stability. Food Hydrocoll..

[56] Zhang W., Xu X., Zhao X., Zhou G. (2022). Insight into the oil polarity impact on interfacial properties of myofibrillar protein. Food Hydrocoll..

