# Supplementary material for: Emulsifying Properties of Oat Protein/Casein Complex Prepared Using Atmospheric Cold Plasma with pH Shifting
Source: Foods. 2025 Jul 31;14(15):2702. doi: 10.3390/foods14152702 (PMC12346296; doi:10.3390/foods14152702)
Supplement: Supplementary file 1 [file foods-14-02702-s001.zip › foods-3678577-supplementary.pdf]

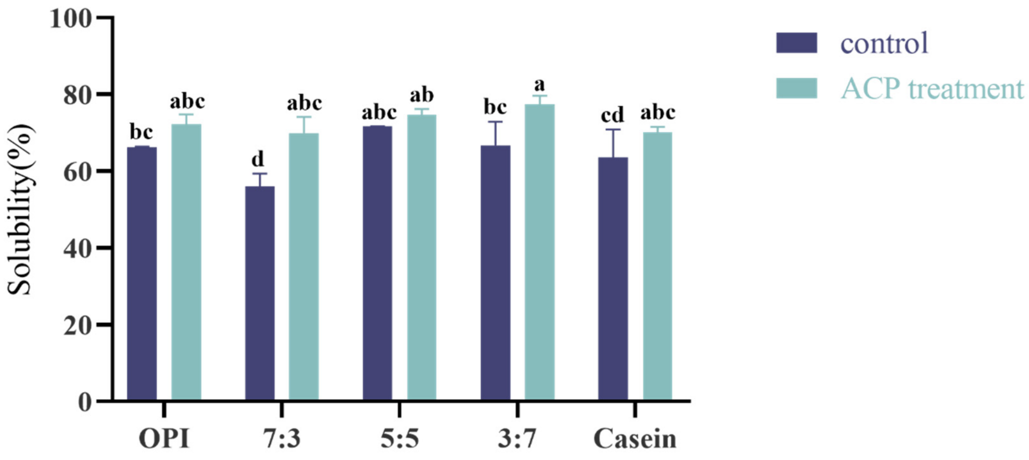

Figure S1: Effect of plasma treatment on the solubility of OPI/casein of different ratios. Different letters indicate significant differences (P < 0.05) among the groups.

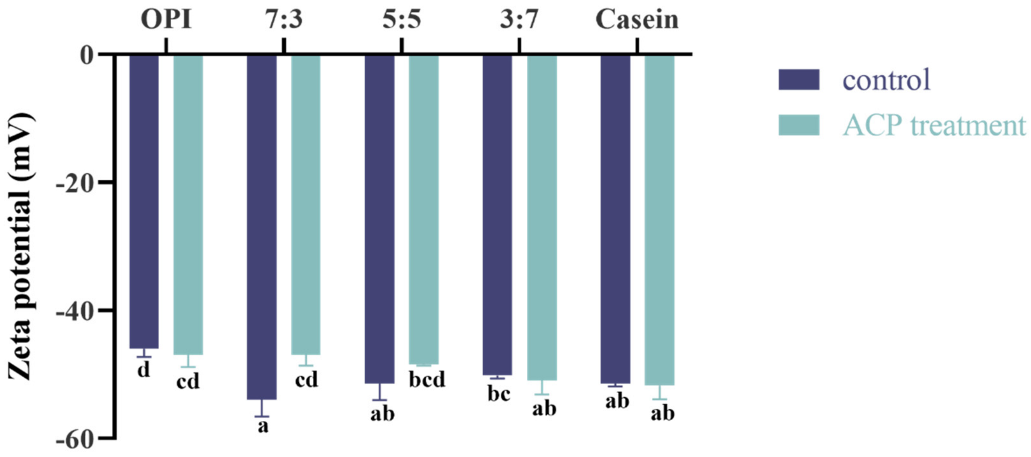

Figure S2: Effect of plasma treatment on the zeta potential of OPI/casein of different ratios. Different letters indicate significant differences (P < 0.05) among the groups.
